# Sentiment Analysis of Customer Reviews of Food Delivery Services Using Deep Learning and Explainable Artificial Intelligence: Systematic Review

**DOI:** 10.3390/foods11101500

**Published:** 2022-05-21

**Authors:** Anirban Adak, Biswajeet Pradhan, Nagesh Shukla

**Affiliations:** 1 Centre for Advanced Modelling and Geospatial Information Systems (CAMGIS), School of Civil and Environmental Engineering, Faculty of Engineering & IT, University of Technology Sydney, Sydney, NSW 2007, Australia; anirban.adak@student.uts.edu.au (A.A.); nagesh.shukla@uts.edu.au (N.S.); 2 Center of Excellence for Climate Change Research, King Abdulaziz University, P.O. Box 80234, Jeddah 21589, Saudi Arabia; 3 Earth Observation Centre, Institute of Climate Change, University Kebangsaan, Malaysia, Bangi 43600, Malaysia

**Keywords:** sentiment analysis, food delivery services, deep learning, explainable artificial intelligence, lime, shapley

## Abstract

During the COVID-19 crisis, customers’ preference in having food delivered to their doorstep instead of waiting in a restaurant has propelled the growth of food delivery services (FDSs). With all restaurants going online and bringing FDSs onboard, such as UberEATS, Menulog or Deliveroo, customer reviews on online platforms have become an important source of information about the company’s performance. FDS organisations aim to gather complaints from customer feedback and effectively use the data to determine the areas for improvement to enhance customer satisfaction. This work aimed to review machine learning (ML) and deep learning (DL) models and explainable artificial intelligence (XAI) methods to predict customer sentiments in the FDS domain. A literature review revealed the wide usage of lexicon-based and ML techniques for predicting sentiments through customer reviews in FDS. However, limited studies applying DL techniques were found due to the lack of the model interpretability and explainability of the decisions made. The key findings of this systematic review are as follows: 77% of the models are non-interpretable in nature, and organisations can argue for the explainability and trust in the system. DL models in other domains perform well in terms of accuracy but lack explainability, which can be achieved with XAI implementation. Future research should focus on implementing DL models for sentiment analysis in the FDS domain and incorporating XAI techniques to bring out the explainability of the models.

## 1. Introduction

Customer satisfaction is the key in assessing how a product or service of a company meets customer expectations [1] and is an important tool that can give organisations major insights into every part of their business, thus helping them to increase earnings or minimise marketing expenses [2]. Customer feedback might help in reviewing the factors that were not previously considered, such as shipping, safe packing, politeness and available customer service consultants and a user-friendly website. Nothing can make customers feel that they are important than asking for their views and valuing their comments. When a customer is asked for any opinion on a product or experience, they feel valued and connected to the organisation [3]. In the food industry, customers often look into restaurant reviews before placing their orders. Nowadays, restaurants or food delivery services (FDSs) have a review or feedback system that is integrated in their portal or social media platforms; however, only a few act on customer opinions due to the presence of a large amount of review data across various platforms and the lack of customer service consultants that will go through each of these comments and act on them [4]. At present, organisations need not depend on customer service consultants to read all the reviews because they can rely on artificial intelligence (AI) to solve their problems and save costs.

With the rise of online food delivery marketplaces after the COVID-19 pandemic, FDSs have brought versatility and a variety of restaurants to the comfort and convenience of homes and offices [5]. The increase in immigration from different countries has also given rise to new cuisines being introduced into the country. Customers are provided with a wide range of meal options and the ability to order from the best eateries or restaurants in town while inside their home or office. With applications becoming a standard utility on mobile devices and the global positioning system (GPS) made available to all, the delivery of food to a customer’s exact location is no longer an issue. Customers can track the progress of their order from the time of order until it arrives at their door. With the rising demand for food takeaway services, many digital marketplace platforms are jumping on the bandwagon.

Global ordering and delivery marketplace platforms, such as UberEATS, Deliveroo and Menulog [6], operate in a cost-intensive business model but take responsibility for the entire delivery logistics. These companies offer a complete sales solution to the restaurants and food business owners at no extra cost and work on a commission-based model. With a few taps on the phone by the customer, FDS applications receive orders, pick up the food from restaurants and deliver it to the customer. Customers have various food options from a chain of restaurants. Online food companies are delighted to find out that customers are eager for such services. Amidst projections that Australia’s food delivery industry would grow [7], COVID-19 lockdowns and quarantines have led to an increase in FDSs [8], including third-party apps such as UberEATS, Deliveroo and Menulog, because people are forced to order online while restaurants are closed. With the corresponding increase in orders and feedback, most companies want to effectively use the data to determine the areas for improvement to enhance customer satisfaction.

Customer sentiment can be found in blog posts, comments, reviews or tweets that mention the quality of food, service, delivery time and other details [9]. FDS organisations can understand what customers are saying and perceive positive comments as compliment and negative comments as complaints [10]. The negative sentiments can be classified into various complaint categories using topic modelling. [11] Customer experience with food can vary with different seasons, such as the increase in positive feedback during the peak season. Despite huge revenues and investments, FDS organisations still struggle with profitability due to high expenses. Predatory pricing is a commonly used strategy to beat the competitive market where businesses swallow a sales loss by massively subsidising meal costs. Furthermore, online FDSs have minimal control over food quality which is highly dependent on the restaurants. If a customer is dissatisfied with the quality, then the food delivery company needs to bear the revenue loss. As a result, businesses such as Sprig [12] and Munchery [13] are unable to endure the loss of revenue and have exited the business [14]. Tracking customer reviews and feedback is the only way for food delivery companies to ensure that the customer experience of the delivery operation is good and does not damage the dine-in experience.

**Figure 1:**
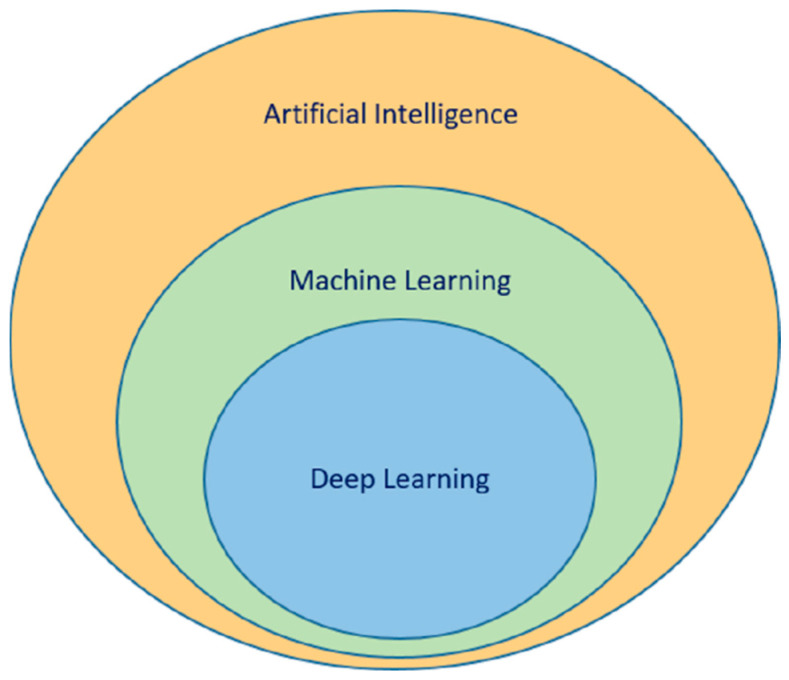
High-level Artificial Intelligence diagram.

The use of AI in natural language processing (NLP) has immense potential to determine positive, negative and neutral reviews [15]. Machine learning (ML) and deep learning (DL) techniques are often used interchangeably in AI but have different meanings. At a high level, ML automates analytical model building, and [16] DL is the subset of ML (see Figure 1) concerned with algorithms inspired by the structure and function of the brain called artificial neural networks.

By realising the importance of customer feedback complexed by a large volume of customer review data and the success of AI in improving prediction accuracy in other fields, FDS organisations can automate the process of predicting customer sentiment and work towards improving the issues. In selecting ML models, a trade-off always exists between accuracy and interpretability. For instance, the black-box DL model produces high accuracy but often lags on interpretability because of the difficulty in explaining the rationale behind the decisions made. Explainable artificial intelligence (XAI) promises to resolve the issue of explainability and interpretability of DL black boxes [17].

With the continuously increasing volume of customer review data, a robust end-to-end framework using AI/ML can help accurately predict customer sentiment. Such a framework will be beneficial for FDS organisations, such as Ubereats, Menulog and Deliveroo. The explanations of ML-based black box models will help build the trust in the system. The solution to predict the sentiment of customer reviews in FDS domain has evolved from lexicon methods to ML and DL. Several papers [18,19,20,21] have presented the sentiment analysis of customer reviews using lexicon-based, ML and DL techniques in the FDS domain; however a review on DL methods or XAI techniques in the same domain is lacking.

To fill this gap, this systematic review was conducted on studies using DL and XAI methods to detect customer sentiments from their reviews and interpret the DL model. This study will benefit FDS organisations by allowing them to identify and resolve customer negative reviews, which will in turn increase customer satisfaction.

## 2. Background

Customer management, an important factor in the FDS business, is measured with customer engagement. Retaining customers becomes extremely crucial when the market is competitive and the company desires to improve the FDS [18]. The first step in customer engagement is to receive feedback and reviews. Feedback acts as a learning tool that makes customers feel important and valued. A business needs to rectify its limitations for an enhanced takeaway home delivery system by analysing genuine feedback from customers. Sentiment analysis is the information that comes directly from the customers about their overall experience and opinion about a business, product or service [19]. The experience can be in the form of satisfaction or dissatisfaction and may be positive, negative or neutral [19]. Also known as opinion mining, sentiment analysis has gained importance over time due to the steep increase in the amount of customer feedback available online in the form of tweets or reviews [20]. People share their opinions on restaurants and food on social media and make their comments visible to any person on the internet. Feedback helps customers decide on product purchases. A large amount of positive feedback from customers increases the chances of selling the product and attracts attention in the market. Sentiment analysis is also important for businesses and decision-makers [19] because it provides market insights that help companies identify the key areas in improving customer experience and their brands. When a customer orders food online using websites or mobile apps, a pop-up window appears asking for feedback, thus greatly increasing customer engagement. When customers plan to order food online, they prefer to look for accurate reports [10]. If the FDS app or website does not have any online reviews, then customers may change their decision to order. Having ‘no reviews’ can be just as detrimental as having negative reviews. Having genuine and positive reviews helps increase the credibility factor. Negative reviews are difficult to handle for any business. They can drive potential customers away from the FDS and prompt existing customers to question whether they want to re-order. Thus, FDS operators have to remember that they cannot control every customer’s experience, mistake or circumstance. On the bright side, a negative review can provide insights into the customer service’s weaknesses and provide opportunities for its improvement [21].

The key benefits of sentiment analysis [20] for business are as follows:

- Keeps businesses connected round the clock with the customers;
- Provides business insights to help in decision-making;
- Indicates real-time trends with emotion data;
- Helps improve the business plan of action to gain an advantage over competitors;
- Can be conducted on services or products to understand which item is eliciting negative sentiments;
- Provides a great tool for businesses to improve customer service in any domain.

## 3. Methodology

A standard review process can be described in three steps: plan, conduct and report [22].

**Step 1:**  Review planning, which is crucial due to the following reasons:

- COVID-19 has increased the demand for online FDSs;
- Improving customer satisfaction and meeting customer expectations;
- Challenges in the adaptation of DL methods for sentiment analysis due to the reduced explainability of models.

The first step was divided into various sections such as ‘Aim and research question’, ‘Search and selection process’, ‘Inclusion and exclusion criteria’, ‘Quality assessment’ and ‘Data extraction and synthesis’.

**Step 2:**  A review phase was conducted by searching and identifying relevant journals and articles with the following keywords: ‘sentiment analysis of customer reviews’, ‘food’, ‘deep learning’, ‘machine learning’, ‘explainable AI’, ‘XAI’, ‘natural language processing’ and ‘food delivery services’ from Scopus database. This review focused on different ML and DL techniques used in customer sentiment analysis in FDS and selected papers on XAI, DL model and NLP task. A total of 97 papers published from 2001 to 2022 were found and considered for the aforementioned task. Step 2 is described in the ‘Results’ section.
**Step 3:**  The report phase involves a discussion of the findings, assessment, recommendations and conclusions identified from the research and review papers. This review concludes with the future research direction of increasing the accuracy and explainability of DL models with the help of XAI. Step 3 is placed under ‘Section 5 and Section 6’.

### 3.1. Aim and Research Questions

The key motivation for this work is as follows. Studies on the sentiment analysis of FDS showed the usage of data mining and ML techniques but lacked focus on DL methods. Additionally, organisations require decision-making models which are justifiable and legitimate. However, no comprehensive study has been conducted to provide insights into the interpretability of published research and the application of state-of-the-art XAI techniques in the FDS domain.

The objectives of this review are to identify the DL techniques applied in the FDS domain for the sentiment analysis of customer reviews, determine the interpretability of published research, identify XAI techniques applied in the FDS domain to bring out the explainability of the models and answer the following questions:

- What are the different AI methods used in the sentiment analysis of customer reviews for FDS?
- Is the research on DL technique adequate to identify the negative sentiments of customer reviews?
- What are the challenges in using DL techniques for businesses?
- Can XAI techniques provide explanation and build trust in the DL model?

### 3.2. Search and Selection Process

Table 1 describes the keywords (food, deep learning, machine learning, natural language processing, food delivery services, online food delivery and XAI) used to search the Scopus library. The keyword search criteria were ‘Search within: Article title, Abstract, Keywords’. Only published and peer reviewed papers were considered for further review. After the list of papers from the search results was skimmed, the papers were classified into four categories as shown in Table 2.

Among the 95 papers, 40 were classified as duplicate from different search queries and hence were excluded from further review. Additionally, 25 papers were found to be generally related to the FDS domain, and a few were referred to establish context as necessary. These papers were searched and retrieved separately from the University of Technology Sydney library, internet and organisation websites.

## 4. Results

Sentiment analysis can be characterised into two primary classifications: lexicon-based and ML/DL methods. Lexicon-based methods [23] use data dictionaries, such as SentiWordNet [24] and SenticNet [25], to tag words as either positive or negative, and the entire sentiment of the sentence is evaluated by summarising the tagged words. The lexicon-based approach is classified under the unsupervised method which does not deal with the polarity labels of the datasets. Given that these methods depend on lexicons, their prediction varies in different domains. Additionally, the sentiments derived in one domain may not be applicable to another domain because various domains have different meanings [26]. For example, ‘lightweight’ in kitchen appliances may provide a negative sentiment, but the same description in electronics and mobile appliances will provide a positive result. To overcome this issue, FDS organisations need to use a cross-domain sentiment adaptation ML/DL classifier that is applicable to any domain.

DL models which comprise hundreds of layers and parameters outperform traditional ML algorithms in sentiment classification and review rating prediction [27] but are still considered as complex as a black box. Additionally, FDS organisations need models capable of making decisions which are justifiable, legitimate and can explain the behaviour. Although DL models bring accurate results [28], they are often criticised for being non-transparent and having predictions that are untraceable by humans [17]. Explainable artificial intelligence (XAI) promises to resolve the issue of explainability and interpretability of DL black boxes [17].

During the last two decades, the World Wide Web (WWW) has emerged as the world’s most important source of information, containing an enormous amount of human-generated reviews on products and services [29]. It is nearly impossible for any FDS organisation to read and analyse all of these positive or negative reviews manually and categorise them into similar classes. Different topic modelling techniques can be explored to categorise sentiments into similar classes to solve this problem [19,29,30]. In topic modelling, each aspect is considered as a topic that has a correlation with a particular domain [29]. Since only those topics that exist in the review could only be identified, these topics are expressed in explicit words. For example, in this sentence, “The phone is great but the battery life is short”, there are two aspects (phone and battery) which will picked by topic modelling. Both the words were present in the sentence and hence such kind of topic is easier to find. On the other hand, in this sentence “It is light as feather”, there is no explicit word which represents aspect and tells the sentence is talking about weight. Topic modelling techniques cannot identify these implicit aspects because there is no word in this review that could be a potential topic [29]. In customer reviews, there could be various words used for the same aspect. For example, in the case of a mobile phone, LCD and screen both refer to the same thing. Picture and movie are synonyms in the movie domain, but they do not represent the same thing in the camera domain, where they are two different things. Photo and picture are again interchangeable terms in the camera world. Traditional dictionary-based approaches do not work well, whereas topic modelling in these scenarios can group similar items into topics. As a result, topic modelling has demonstrated its utility in grouping related topics [31].

Common FDS customer complaint types found in published papers are presented in Table 3.

The FDS common complaint types described in Table 3 can be categorised into four common groups (delivery time, customer service, food quality and cost) [19,37] as shown in Table 4. Organisations can channel the concerned department to address the issues and increase customer satisfaction to promote their brand or product.

There have been several comprehensive and systematic review papers published over the decade that describe topic categorisation using various techniques applied in sentiment analysis; hence, this paper will not describe those methods again and instead focus more on ML/DL and XAI techniques. This section describes previous findings on implementing ML and DL models for sentiment analysis in the FDS domain. For the explainability and interpretability of ML and DL models, the implementation of XAI techniques in other domains was discussed in the XAI section.

### 4.1. ML Techniques

Several comprehensive and systematic review papers on ML applied in various domains have been published. The present systematic review describes those methods and will only focus on the ML techniques used in the FDS domain. A review [38] on customer review analytics on FDS in social media used AI algorithms and methods to perform sentiment analysis on FDS. Four different AI algorithms, namely, lexicon, support vector machine (SVM), NLP and text mining, were analysed and compared. Lexicon achieved the highest accuracy of 87.33%, followed by NLP at 71.67%, SVM at 69.70% and text mining at 67.94%. Therefore, the lexicon-based approach works better than ML algorithms (SVM).

A systematic review on sentiment analysis in social media and its application [39] revealed that the two main methods of sentiment analysis are ML and lexicon-based approaches. The former detects sentiment from data using its algorithm, and the latter uses positive and negative words from the sentence. Various AI methods have been introduced by researchers, but the most commonly used methods are still SentiWordnet and TF-IDF for lexicon-based approaches and Naïve Bayes and SVM for ML approaches [39]. Despite their higher accuracy than ML models, lexicon-based approaches are challenging to use in sentiment analysis in languages other than English.

A recent work [40] performed sentiment analysis on movie review data and found that ML models (Naïve Bayes, maximum entropy classification and SVM) do not perform well on sentiment classification compared with traditional topic-based categorisation. The key gap was that the models cannot achieve accuracies on sentiment classification problem compared with standard topic-based categorisation. The researcher gave an example of the sentence ‘How could anyone sit through this movie’ which contains no negative word.

According to the above literature, lexicon approaches provide higher accuracy and are more frequently used compared with ML models. However, challenges arise in performing sentiment analysis in languages other than in English. Additionally, a gap exists where the entire sentence can have negative sentiment without having any negative word [26]. Domain adaptation is another aspect which needs to be considered while building models; words in one domain can have different meanings in another domain. Additional research work is required to address the above gaps in the FDS domain.

### 4.2. Deep Learning

Ref. [27] indicated the success of DL models which comprise hundreds of layers and parameters and outperform traditional ML algorithms in sentiment classification and review rating prediction. Some challenges arise with DL usage, such as the requirement for large data, heavy computing and training models. Nevertheless, in today’s world, these challenges are no longer an issue because of the availability of high-performance computing facilities.

#### 4.2.1. Recurrent Neural Network (RNN)

RNN is a class of neural networks which works well with a sequence of data input [41]. NLP tasks, such as sentiment analysis, can be easily solved by RNN. Different from traditional neural networks, RNN can remember the previous computation of information and can apply it to the next sequence of inputs.

According to some researchers [33,42], DL algorithms (bidirectional long short-term memory (Bi-LSTM) and simple embedding and average pooling) outperform traditional ML algorithms in sentiment classification and review rating prediction. They proposed the use of DL technique during the COVID-19 pandemic to help customers in making safe dining decisions. The review data were obtained using a web scraper from Yelp restaurants located in the top 10 cities by population in the United States and were pre-processed by tokenisation and stopword removal [34,43]. Term frequency-inverse document frequency was used to identify the key features from the reviews and place them into meaningful categories. The results showed that the bidirectional LSTM algorithm is effective in generating subtopics and sentiment prediction, and the simple embedding and average pooling performs well in online review prediction tasks. In [33], it was suggested that RNN models require a high level of supervision and that future works should focus on the bidirectional RNN model.

A systematic review on sentiment analysis in social media conducted by [39] revealed that RNN has a longer computational time than other DL models (convolution neural network, CNN). Common DL models such as RNN, LSTM and CNN have been individually tested in different datasets; however, their comparative analysis is lacking. Ref. [34] highlighted that DL models such as RNN is efficient in handling a large volume of complex data but is often criticised for being a black-box model. Further work must be conducted for the comparative analysis of DL models in performing sentiment analysis in the FDS domain.

#### 4.2.2. CNN

CNN is widely popular because it can be used in image datasets by extracting the significant features of the image while the ‘convolutional’ filter (i.e., kernel) moves through the image [44]. CNN could also be used in text with 1D input data [45]. While the filter moves in the text area, the local information of texts is stored, and important features are extracted. Hence, CNN can be effectively used for text classification. Kim [28] found that CNN models outperformed previous approaches for several classification tasks. With the slight tuning of the hyper-parameters, one-layered CNN performs remarkably well. Moreover, unsupervised pre-training of word vectors plays a key role in DL for NLP. Bhuiyan et al. [38,46] found that attention-based CNN model had the highest accuracy of 98.5% compared with that of baseline CNN at 96.34% and LSTM at 97.23%. They proposed to work on the usage of bidirectional encoder in the FDS domain because it produces the best results with extremely long training time compared with CNN. Hung [47] indicated that the hybrid model of CNN with LTSM is more accurate than CNN or LTSM. The accuracy of the hybrid model is 83.45%, whereas that of individual CNN and LSTM is 82.76% and 82.54%, respectively. Muhammad et al. [48] compared the performance of various ML algorithms such as SVM, logistic regression, random forest and NB and found that the CNN model outperformed all ML algorithms. Therefore, CNN can be used in text mining tasks with high accuracy and could be applied for customer sentiment analysis on FDS.

According to the literature, hybrid DL models should be tried to attain accuracy in performing sentiment analysis. Additional research must be conducted to improve the interpretability of the black box models of DL algorithms.

### 4.3. XAI

Arrieta et al. [49] noted the success of DL models which comprise hundreds of layers and parameters considered as a black box. Organisations need models capable of making decisions which are justifiable and legitimate. A common perception is that if the model only targets accuracy and performance, then the system would become opaque. However, understanding the model features would enable the improvement of its deficiencies. According to Singh et al. [50], DL is significant in medical diagnostic tasks and outperformed human experts. However, due to the black-box nature of the algorithm, it is not being used across the industry. Wolanin et al. [51] signified the importance of ML and DL in the context for forecasting crop yields (different domains) but added that these algorithms lack transparency and interpretability. The black-box nature of DL restricts its usage across the industries because it lacks trust and explainability.

Interpretability is the degree to which a human can comprehend the reason for the model’s outcome [43]. Deep neural networks lack interpretability, and the model features that drive the outcome are difficult to understand [17,52,53,54,55,56,57]. XAI or interpretable machine learning IML programs aim to produce explainable models while maintaining a high level of accuracy. Schoenborn and Althoff [58] indicated that the need for explainable AI has increased rapidly due to the increase in usage of DL and recent legal restrictions. The goal is to bring people to trust AI which can be achieved through explainable AI. In implementing DL models, we need to provide explainability on how the model predicts its outcome so that industries and organisations can build trust to apply the black-box model. A possible scenario is that a DL model has extremely high accuracy for wrong reasons and organisations cannot trust any model without knowing which feature or dimension served as the basis of the prediction. However, Mathews [59] mentioned that black boxes should not be used in critical systems such as medical field or malware detection because wrong decisions can result in harmful consequences.

Most research in FDS achieved accuracy with non-interpretable models. Table 5 shows the recent papers on sentiment analysis in FDS with model interpretability.

Table 5 shows that 45% of the papers used a model built on DL and 55% used a model built on ML. The key fact is that 77% of the models are non-interpretable in nature; hence, organisations can argue for the explainability and trust in the system. No study has been conducted on XAI with DL on NLP for sentiment analysis across the FDS industry, which represents a scope for future research. Many XAI methods can be applied to DL models to increase the explainability component and ensure high accuracy. The most popular two XAI methods are the following.

#### 4.3.1. Local Interpretable Model-Agnostic Explanations (LIME)

Shankaranarayana and Runje [62] proposed a method called LIME [63]. LIME is one an XAI technique that generates single-instance level explanation by artificially generating a dataset around the instance (by randomly sampling and using perturbations) and then training a local linear interpretable model. For sentiment analysis, organisations need to understand the words or features which contribute greatly in predicting the reviews to be negative, neutral or positive. Given the previous application of LIME in other domains [59,64], it can be used in DL models to analyse customer reviews in the FDS domain. No research has been published on sentiment analysis in FDS and DL along with LIME interpretability.

#### 4.3.2. Shapley Additive Explanation (SHAP)

SHAP [65] is based on the principle of adding the SHAP value as a contribution to all the variables of a data point to derive the final outcome. This technique functions in the same way as any team sport, such as cricket or football. Once a cricket match is completed, post-match analysis can be performed using a SHAP-based algorithm. For any outcome such as win, lose or draw, contributions from all 11 players can be used to evaluate the SHAP value for each player. Internally, SHAP uses Kernal SHAP method from [66], which computes the weight as a contribution for all the features of the black box. SHAP is built to enhance the features of LIME. Different from that in LIME, a local linear module is not built in SHAP. Instead, some functions are used to calculate the shapely value. In sentiment analysis, the SHAP algorithm can be used to determine the contributions of each word towards positive and negative sentiment. However, no research has been conducted on sentiment analysis in the FDS domain and DL along with SHAP interpretability.

#### 4.3.3. Comparison of LIME and SHAP

The major difference between LIME and SHAP is that the LIME value is evaluated by removing the variables or features to obtain an outcome, and the SHAP value is the contribution of all the variables or features to make a prediction [67]. Owing to this nature, LIME is much faster than SHAP because the latter considers all the possible combinations of the variables with contributions to create the outcome.

## 5. Discussion

This study showed that the performance of ML models (Naïve Bayes, maximum entropy classification and SVM) on sentiment classification is not as good as that of traditional topic-based categorisation [40]. Customer reviews can be negative without having any negative word in the sentence. Additionally, lexicon-based approaches can achieve higher accuracy than ML models but are challenging to implement in sentiment analysis in languages other than in English [26]. Domain adaptation is another aspect which must be considered in building models because the same words can have different meanings in another domain. The mentioned challenges may be solved by using DL algorithms where the model trains itself from a large chunk of data from the same domain.

DL methods such as RNN, CNN, and LSTM showed good performance. However, further experiment and research must be conducted on hybrid approaches where multiple models and techniques are combined to enhance the sentiment classification accuracy [68]. Although neural networks provide high prediction accuracy [28], they lack explainability. Owing to the opaqueness of the DL techniques, businesses are reluctant to use black-box models and prefer to verify and check how the models are predicting accurate results. XAI techniques such as SHAP and LIME can support DL techniques in explaining how the model is determining the correct customer sentiment of a review. LIME and SHAP results can be compared with those from DL techniques.

By performing sentiment analysis using the DL/ML methods on customer reviews, FDS organisations can use the data to analyse customer complaints and work towards improving customer satisfaction. The output customer review data from DL/ML model is labelled as negative and positive sentiment. The ML/DL model is verified using the XAI technique against its computing logic. As topic modelling can group related topics, the negative sentiments can be grouped into different classes (delivery time, customer service, food quality and cost) as shown in Table 4. FDS organisations can use this information to understand which particular group class is getting more problem. Different problem categories may be sent to the respective team. If the negative sentiments are due to an increase in delivery time, then organisations may need to solve their supply-chain-related problems. FDS organisations may also look into logistics issues by determining the number of vehicles and delivery boys needed when delivering to far-off destinations. In case of large orders in restaurants, delivery time sometimes increases due to larger wait time. The higher delivery time data may be further grouped upon location to check if the problem is happening for some locations or all locations. If the negative feedback comes under customer service category, then the service level must be paid attention. With food delivery, there is always a risk of poor packaging or spillage and hence food quality issues must be resolved at the respective restaurants, and organisations can keep an eye on the restaurants which are contributing to negative reviews due to food quality. Complains on the cost of the food item can be resolved by the restaurant and the organisation by reducing the cost or lowering the profit margin. Several other complaint groups can be considered by the FDS organisation to solve their customer feedback complaints. Topic categorisation on positive sentiments can also be used to reward staffs or restaurants. FDS organisations may think of more meaningful topic groups based on their business requirement. Although topic modelling has performed significantly well in topic categorisation, but there is a need to compare these techniques on the FDS domain.

### 5.1. Findings of the Study

Compared with ML techniques, DL is more accurate in predicting customer sentiment analysis. Given that deep neural networks are black-box in nature, DL models need support from XAI techniques, such as LIME or SHAP, to explain the features on which algorithms are computed to ensure high accuracy and explainability and earn the trust of businesses. The combination of DL with XAI on FDS would help in understanding the customer sentiments about food and service quality worldwide and subsequently improving customer satisfaction. Furthermore, topic modelling can be conducted on customer reviews to categorise them in meaningful groups. According to the volume of complaints in each group, organisations can prioritise their action and send it to the right channel for a solution.

### 5.2. Future Prospects

**Figure 2:**
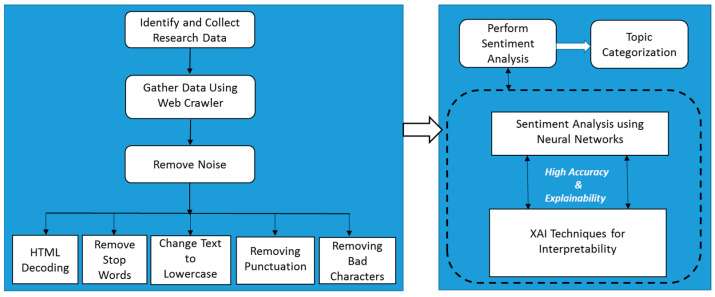
Proposed diagram for future work with high accuracy and explainability.

Although customer feedback or reviews are easily obtained from blog posts, comments, reviews or tweets, the data can be of a very large volume. DL models have always shown good performance with a large volume of data. Thus, new DL or hybrid models should be tested to obtain the best accuracy. The negative sentiments can be categorised into various complaint groups using topic modelling. For the DL models, explainability must be reduced to achieve high accuracy; however, XAI can support the explainability part of the model. Several research papers have presented the usage of ML or DL techniques for sentiment analysis in customer reviews; however, no study has been conducted on XAI with DL in the FDS domain. With the surge in FDS usage due to COVID-19 lockdowns, the solution (Figure 2) can definitely help the food industry to quickly adapt to customer requirements and preferences.

## 6. Conclusions

This study reviewed and discussed past research works on customer sentiment analysis with ML and DL techniques across the FDS domain. Results showed that DL techniques (CNN, LTSM and Bi-LTSM) have great accuracy but lack explainability; their interpretability can be improved with XAI implementation. Domain adaptation by the models is a key aspect in sentiment analysis. In consideration of the increase in sales and competition across this domain, additional research work is required on sentiment analysis in the FDS domain using DL techniques with XAI. Thus, we recommend the following research directions:

- Further research on the sentiment analysis of customer reviews using DL techniques such as CNN, LTSM and Bi-LTSM and comparison of the results;
- Usage of XAI techniques such as LIME or SHAP to explain and build trust in the DL models from the previous step;
- Classification of negative sentiments into various topic categories using topic categorisation techniques to address supply chain issues and improve customer satisfaction; and classification of the positive sentiments into various topic categories using topic categorisation technique to appreciate or reward employees.

## Figures and Tables

**Table 1:** Search queries and results showing the number of papers.

Number	Search Query	Number of Papers
1	‘Sentiment Analysis of customer reviews’ AND ‘food’	47
2	‘Sentiment Analysis of customer reviews’ AND ‘food’ AND ‘deep learning’	5
3	‘Sentiment Analysis of customer reviews’ AND ‘food’ AND ‘machine learning’	18
4	‘XAI’ AND ‘deep learning’ AND ‘natural language processing’	6
5	‘Sentiment Analysis’ AND ‘ Food Delivery Services’	7
6	‘ Sentiment Analysis’ AND ‘ Online Food Delivery’	8
7	‘XAI’ AND ‘Food’	5

**Table 2:** Literature classification.

Paper Classification	Machine Learning	Deep Learning	Explainable AI Methods	Other Methods	Total
Duplicate papers	18	6	1	15	40
Non-relevant to FDS	9	1	10	10	30
General FDS paper	8	4	0	13	25
Total	35	11	11	38	95

**Table 3:** Common complaint types in FDS.

Complaint Types	References
Service, missing item, problem with order, missing order, rude service	[4,15,19,32,33,34]
Food, food quality, food taste	[4,15,19,32,33,34]
Place, location	[19,27,35]
Experience, environment, ambiance, dining atmosphere	[4,15,27,35,36]
Value for money, restaurant value, cost	[4,15,27,35,36]
Time, slow service, slow delivery	[19,33]

**Table 4:** FDS common complaint categories.

Delivery Time	Customer Service	Food Quality	Cost
Time, slow service, slow delivery	Service, missing item, problem with order, missing order, rude service, place, location, experience, environment, ambiance, dining atmosphere	Food, food quality, food taste	Value for money, restaurant value, cost

**Table 5:** Interpretability of methods used for sentiment analysis in FDS.

No.	Paper	Algorithm	ML/DL	Year	Is Method Interpretable	Refs
1	Comparative study of deep learning models for analysing online restaurant reviews in the era of the COVID-19 pandemic	Bidirectional LSTM and Simple Embedding + Average Pooling	DL	2021	No	[33]
2	Integrating Sentiment Analysis in Recommender Systems	LSTM, CNN, LSTM-LSTM	DL	2020	No	[47]
3	Aspect-based sentiment analysis and emotion detection for code-mixed review	Gated Recurrent Unit (GRU) and Bidirectional Long Short-Term Memory (BiLSTM)	DL	2020	No	[42]
4	An Attention Based Approach for Sentiment Analysis of Food Review Dataset	Convolutional neural networks (CNN)	DL	2020	No	[42]
5	Sentiment analysis and classification of restaurant reviews using machine learning	Naïve Bayes Classifier, Logistic regression, Support Vector Machine (SVM), and Random Forest	ML	2020	No	[36,46]
6	‘How was your meal?’ Examining customer experience using Google maps reviews	Logistic regression	ML	2020	No	[32]
7	Aspect-based Opinion Mining for Code-Mixed Restaurant Reviews in Indonesia	Logistic regression, Decision tree	ML	2019	No	[42]
8	Sentiment Analysis of Bengali Texts on Online Restaurant Reviews Using Multinomial Naïve Bayes	Multinomial naïve Bayes	ML	2019	Yes	[60]
9	An Experimental Study of Supervised Sentiment Analysis Using Gaussian Naïve Bayes	Gaussian naïve Bayes	ML	2018	Yes	[61]

## Data Availability

Not applicable.
